# Pharmacological and Genetic Evidence of Dopamine Receptor 3-Mediated Vasoconstriction in Isolated Mouse Aorta

**DOI:** 10.3390/biom11030418

**Published:** 2021-03-11

**Authors:** Veronica Zingales, Sebastiano Alfio Torrisi, Gian Marco Leggio, Claudio Bucolo, Filippo Drago, Salvatore Salomone

**Affiliations:** Department of Biomedical and Biotechnological Sciences, University of Catania, via S. Sofia 97, 95123 Catania, Italy; vezin@uv.es (V.Z.); sebastiano.torrisi@unict.it (S.A.T.); gianmarco.leggio@unict.it (G.M.L.); bucocla@unict.it (C.B.); f.drago@unict.it (F.D.)

**Keywords:** mouse aorta, vasoconstriction, dopamine receptors, 7-OH-DPAT, SB-277011-A, L741,626

## Abstract

Dopamine receptors (DRs) are generally considered as mediators of vasomotor functions. However, when used in pharmacological studies, dopamine and/or DR agonists may not discriminate among different DR subtypes and may even stimulate alpha1 and beta-adrenoceptors. Here, we tested the hypothesis that D2R and/or D3R may specifically induce vasoconstriction in isolated mouse aorta. Aorta, isolated from wild-type (WT) and D3R^−^/^−^ mice, was mounted in a wire myograph and challenged with cumulative concentrations of phenylephrine (PE), acetylcholine (ACh), and the D3R agonist 7-hydrxy-N,N-dipropyl-2-aminotetralin (7-OH-DPAT), with or without the D2R antagonist L741,626 and the D3R antagonist SB-277011-A. The vasoconstriction to PE and the vasodilatation to ACh were not different in WT and D3R^−^/^−^; in contrast, the contractile responses to 7-OH-DPAT were significantly weaker in D3R^−^/^−^, though not abolished. L741,626 did not change the contractile response induced by 7-OH-DPAT in WT or in D3R^−^/^−^, whereas SB-277011-A significantly reduced it in WT but did not in D3R^−^/^−^. D3R mRNA (assessed by qPCR) was about 5-fold more abundant than D2R mRNA in aorta from WT and undetectable in aorta from D3R^−^/^−^. Following transduction with lentivirus (72-h incubation) delivering synthetic microRNAs to specifically inactivate D2R (LV-miR-D2) or D3R (LV-miR-D3), the contractile response to 7-OH-DPAT was unaffected by LV-miR-D2, while it was significantly reduced by LV-miR-D3. These data indicate that, at least in mouse aorta, D3R stimulation induces vasoconstriction, while D2R stimulation does not. This is consistent with the higher expression level of D3R. The residual vasoconstriction elicited by high concentration D3R agonist in D3R^−^/^−^ and/or in the presence of D3R antagonist is likely to be unrelated to DRs.

## 1. Introduction

Dopamine (DA) is a catecholamine neurotransmitter involved in a variety of functions, including locomotor activity, cognition, motivation and reward, food intake, and endocrine regulation. The DA transmission in central nervous system (CNS) has been highly studied, mainly because its dysfunction is implicated in severe pathological conditions, such as schizophrenia, Parkinson’s disease, and Tourette’s syndrome.

DA exerts its action by binding to five distinct seven transmembrane domain/G-protein coupled receptors, grouped in two subfamilies, D_1_- and D_2_-like, on the basis of their biochemical and pharmacological properties [1]. D_1_-like receptors, comprising D_1_ and D_5_ receptors (D1R, D5R), mainly activate adenylate cyclase activity; D_2_-like receptors, comprising D_2_, D_3_, and D_4_ receptors (D2R, D3R, D4R), mainly inhibit adenylate cyclase activity and regulate some ionic channels. D1R is the most abundant subtype in the CNS; D5R is found at a much lower level than D1R, mainly in the hippocampus and thalamus. D2R and D3R are expressed in the striatum, olfactory tubercle, islands of Calleja, nucleus accumbens, substantia nigra pars compacta, ventral tegmental area, and the pituitary gland. D2R and D3R are pre- and post-synaptic, unlike D1R and D5R, which are mainly post-synaptic receptors. D4R is found in the basal ganglia and in the frontal cortex, medulla, amygdala, hypothalamus, and mesencephalon, at levels below the other DRs [1,2]. DRs may also form dimeric and/or oligomeric complexes by association of a single species (homodimer, homomer) or different species (heterodimer, heteromer) [3]. Genetic and pharmacological studies have demonstrated the central role of D2R and D3R in the pathophysiology and treatment of schizophrenia, Parkinson’s Disease, drug addiction, and depressive disorders. The first-line pharmacological treatment for schizophrenia is represented by antipsychotics, considered D2R antagonists, and later on reconsidered as D2R-like antagonist, to indicate their poorly selective binding at D2R, D3R, and D4R [4]. An imbalance favoring increased D2R signaling in the prefrontal cortex is associated with improved executive and working memory [5,6]. Current pharmacological treatments for Parkinson’s disease aims at restoring dopaminergic transmission by using L-DOPA, the precursor of DA, or DR agonists, such as bromocriptine, rotigotine, pramipexole, and ropinirole [7]. Another field of interest in DR studies is addiction. D3R are involved in drug-related reward and intake and in behavioral sensitization, including drug-seeking behavior [8,9]. Furthermore, increased D3R expression has been reported following chronic exposure to psychostimulant drugs [10,11] or ethanol intake [12,13]. Activity of mesolimbic DA neurons in the reward circuit is also a determinant of susceptibility/resilience to chronic stress [14,15]. D3R^−^/^−^ appear more resistant to stressful procedures than WT littermates [16,17], show better performance in the elevated plus maze, and are more sensitive to the anxiolytic effect of diazepam [18,19].

Though abundant in the CNS, DA receptors (DRs) are also found outside the brain. The discovery of the renal vasodilating function of DA led in fact to an extensive collection of experimental and clinical data concerning its potential role in cardiovascular system. Currently, DA itself and fenoldopam are approved in the US and EU as drugs supposedly acting on cardiovascular DRs. Detection of DRs out of CNS, however, has not been paralleled by demonstration of peripheral DA concentrations high enough to activate them. Furthermore, when exogenously administered to sustain blood pressure and heart activity, DA is likely to activate alpha and beta adrenoceptors [20,21], while fenoldopam, a selective D1R agonist, may also act as an antagonist to alpha adrenoceptors [22,23]. In recent years, the molecular characterization of individual DRs has also been paralleled by a more detailed pharmacological characterization, due to the availability, at least as investigational agents, of some relatively selective ligands, particularly receptor antagonists. This progress, on one hand, has produced further insights (e.g., variants of the D2R have been reported to be associated with hypertension); on the other hand, it could produce novel cardio-vascular drugs with selective DR action. For a long time, vascular DRs were mainly characterized through functional and/or radioligand binding studies. Based on these techniques, DRs have been found in the aorta, renal, coronary, pulmonary, mesenteric, and cerebral arteries of several species, including humans [24]. However, ligands not specific and/or selective enough may have generated ambiguous identification of individual isoforms, particularly within a DR subfamily [25]. Later on, DR expression in vessels has been analyzed at the mRNA and/or at the protein level. These data show that D1R-like receptors are mainly expressed in ECs, while D2R-like receptors are mainly expressed in prejunctional sympathetic nerve endings; less information is available about vascular smooth muscle cells (VSMCs), suggesting that DRs do not generally reach an expression level comparable to that of endothelium and/or sufficient to be discriminated from those expressed in sympathetic nerve endings, which are very close to VSMCs. The functions mediated by these DRs remain largely to be determined; it has been suggested that stimulation of D1R-like receptors in ECs may induce endothelium-dependent vasodilatation [26], an effect that may involve the activation of the GTP-binding protein Rac1, while D2R may inhibit Rac1 [27,28,29]. In VSMC, D1R stimulation supposedly induces vasodilatation through the classical Gs/cAMP/PKA signaling pathway [30,31,32]. Even less clear is the expression and the putative function of D2R-like receptors in VSMC. In fact, as mentioned above, D2R-like receptors seem to be mainly expressed in sympathetic nerve endings, e.g., located in the media, interspersed with VSMC, making it difficult to discriminate the cell type harboring them.

In this study, we took advantage of D3R null mice (D3R^−^/^−^) to assess the role of D3R in arterial vasoconstriction. In particular, we used isolated mouse aorta, from wild-type (WT) and D3R^−^/^−^ as an intact, native system, to assess the vasomotor responses to the D3R agonist 7-hydroxy-2-dipropylaminotetralin (7-OH-DPAT), in the presence and in the absence of the selective D3R antagonist SB-277011-A and in the presence and in the absence of the preferential D2R antagonist L741,626. The results show that 7-OH-DPAT induces a vasomotor response, which seems specifically related to the stimulation of D3R because it is reduced in D3R^−^/^−^ and it is reduced by D3R antagonism in WT.

## 2. Materials and Methods

### 2.1. Animals 

D3R null mice (D3R^−^/^−^) and wild-type (WT) littermates (males, 5–7 months old) were fed on standard laboratory chow and were allowed free access to water in an air-conditioned room, at 22 ± 2 °C, with a 12-h light/12-h dark cycle. Mice D3R^−^/^−^ were tenth to twelfth generation of congenic C57BL/6J mice, generated by a back-crossing strategy [33]. Genotypes were identified by PCR analysis of tail DNA as previously described [16]. Aorta was obtained from control, untreated animals, and sacrificed to analyze the brain sample for other studies, for which animal use was approved by the subcommittee for research and animal care at the University of Catania in accordance with guidelines from Italian Ministry of Health. 

### 2.2. Preparation of Vessels and Analysis of Vascular Responses

Mice were killed by CO_2_ asphyxiation. Aorta was removed, put in physiological salt solution (PSS; composition, mM: NaCl, 118; KCl, 4.6; NaHCO_3_, 25; MgSO_4_, 1.2; KH_2_PO_4_, 1.2; CaCl_2_, 1.2; glucose, 10; EDTA, 0.025), cut in segments (2 mm length), and mounted on the 40-μm diameter stainless steel wire of a wire myograph (610 M, Danish Myo Technology, Aarhus, Denmark) for isometric record of contractile force. After mounting, each preparation was equilibrated unstretched for 30 min, in PSS, at 37 °C and aerated with 95% O_2_|5% CO_2_. The normalized passive resting force and the corresponding diameter were then determined for each preparation from its own length–pressure curve, as previously described [34]. Contractile responses were recorded into a computer by using a data acquisition and recording software (Myodaq and Myodata, Danish Myo Technology). After normalization and 30-min equilibration in PSS, the preparations were stimulated with isotonic, depolarizing, KCl-rich solution, in which part of NaCl had been replaced by an equimolar amount of KCl (composition in mM: NaCl, 22.6; KCl, 98.8; NaHCO_3_, 25; MgSO_4_, 1.2; KH_2_PO_4_, 1.2; CaCl_2_, 1.2; glucose, 10; EDTA, 0.025, pH 7.4 at 37 °C) [35]. After washout, the preparations were exposed to cumulative concentrations of phenylephrine (PE, 1 nM–1 μM); once the contractile response had reached a steady state, cumulative concentrations of acetylcholine (ACh, 1 nM–10 μM) were added to the organ bath to assess endothelium-dependent relaxation. After wash out, the preparations were exposed to cumulative concentrations of 7-OH-DPAT (10 nM–10 μM). To investigate the effect of D3R and D2R antagonists on the vasomotor response induced by 7-OH-DPAT, the preparations were pre-incubated for 30 min with SB-277011-A (10 nM, 100 nM) or with L741,626 (10 nM, 100 nM) before exposing to 7-OH-DPAT.

### 2.3. In Vitro Lentiviral Delivery of Synthetic miRNA

A lentiviral vector (Lv pPGK-eGFP-miR-DR, 10^8^ TU/mL, ICM—Plateforme de Vectorologie, Paris, France), coexpressing under the drive of the ubiquitous PGK promoter the eGFP and a miRNA, specifically directed against the mRNA of D2R or D3R, was used to downregulate D2R or D3R in mouse aorta [36]. Following removal and dissection, aorta was cut in rings and placed in a 96-well plate, with Dulbecco’s modified eagle medium (DMEM), containing 100 U/mL penicillin, 100 μg/mL streptomycin (Invitrogen), and 10% fetal bovine serum (FBS). DMEM, penicillin, streptomycin, and FBS (Fetal Bovine Serum) were from Thermo Fisher Scientific (Waltham, MA, US). Aortic segments were then exposed to one of the lentiviruses (approximately 5 × 10^6^ TU in 200 μL DMEM), carrying synthetic miRNA specific for D2R, D3R, or the control, scrambled sequence, for 72 h, in a cell culture incubator, at 37 °C and 5% CO_2_. At the end of the incubation period the arterial segments were mounted in a wire myograph to examine the vasomotor responses. The expression level of DR mRNA following lentivirus-miRNA-induced knockdown was assessed by real-time quantitative polymerase chain reaction (see below).

### 2.4. Analysis of mRNA Expression by Real-Time Quantitative PCR

Total RNA extraction of mouse aortic segments was carried out by TRIzol (Invitrogen, Carlsbad, CA). Single-stranded cDNA was synthesized with SuperScript III (Invitrogen) by random priming. Aliquots of cDNA were amplified in parallel reactions with external standards at known amounts, using specific primer pairs for D3R, the short and the long splice variant of D2R (D2L, D2S), and GAPDH (reference gene). Each PCR reaction (20 μL final volume) contained 0.5 μM primers, 1.6 mM Mg^2+^, and 1 × Light Cycler-Fast Start DNA Master SYBR Green I (Roche Diagnostics, Indianapolis, IN). Amplifications were carried out in a Light Cycler 1.5 instrument (Roche Diagnostics). Quantification was obtained by the ΔCt comparative method.

### 2.5. Drugs and Reagents

Phenylephrine, acetylcholine, 7-OH-DPAT, and L741,626 were from Sigma–Aldrich (St. Louis, MO, U.S.A). These drugs were dissolved at 10 mM in aqueous stock solutions, except L741,266, which was dissolved in ethanol. The stock solutions were further diluted in water or directly in physiological salt solution, as required to reach the final concentration. SB-277011-A was from Tocris (Milano, Italy); it was dissolved at 10 mM in aqueous stock solution and then further diluted as appropriate. 

### 2.6. Statistical Analysis

PCR data were expressed as mean ± SEM. Data in concentration–contraction curves were expressed as a percentage of KCl-induced vasoconstriction against a log molar concentration of drug. Each set of data points was curve-fitted by a non-linear regression, best-fit, sigmoidal dose–response curve, with no constraints using GraphPad Prism (GraphPad Software, San Diego, CA, US). Each curve represents the mean of at least six individual preparations from a minimum of three or more mice. For each set of experiments, n is expressed as number of preparations. Arterial segments from the same animal were represented in the different experimental conditions. For example, we typically cut one aorta of mouse in eight segments, one was incubated with vehicle (control), the other three with the antagonist (SB-277011-A or L741,626), each experiment run in duplicate. Whole curves were compared by two-way analysis of variance (ANOVA). Statistical significance was set at *p* < 0.05. The concentration–response curves to 7-OH-DPAT with or without the antagonist were carried out in parallel, i.e., they represent comparisons between the very same run/challenge. 

## 3. Results

### 3.1. Vasomotor Responses in Aortic Segments Isolated from WT and D3R^−^/^−^

**Figure 1:**
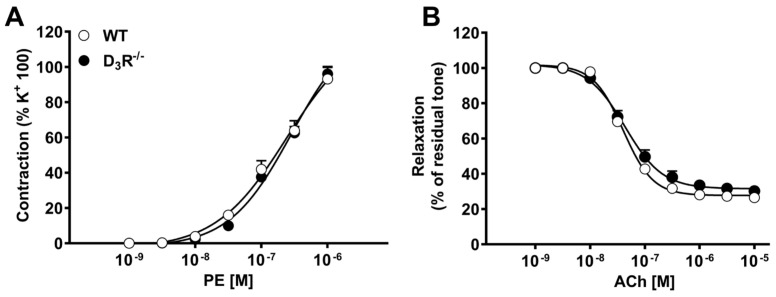
Vasomotor responses in aortic segments isolated from wild-type (WT) and D3R^−^/^−^ mice. (**A**) Vasoconstriction induced by cumulative concentrations of phenylephrine (PE); (**B**) vasodilatation to cumulative concentrations of acetylcholine (ACh). Each curve (with vertical bars representing standard errors) represents the average from 10–12 preparations.

Aortic segments, from either WT or D3R^−^/^−^, were mounted in a wire myograph and first constricted by exposing to a 100 mM KCl-depolarizing solution. After wash out and recovery, the preparations were challenged with cumulative concentrations of the alpha-adrenergic agonist phenylephrine (PE), to induce vasoconstriction, followed by cumulative concentrations of acetylcholine (ACh) to assess endothelium-dependent vasodilatation. The first contraction induced by KCl was not different among the two groups and was further used to normalize the subsequent phenylephrine-induced vasoconstriction, as in previous studies [35]. As shown in Figure 1, no differences were observed in PE-induced vasoconstriction and in ACh-induced relaxation between WT and DR3^−^/^−^. In both groups, the maximum contraction evoked by PE slightly overcame that induced by KCl. Furthermore, ACh relaxed the pre-existing PE-induced tone by about 80%, indicating that functional integrity of endothelium was preserved.

### 3.2. Vasomotor Effect of DR Agonist and Antagonists

**Figure 2:**
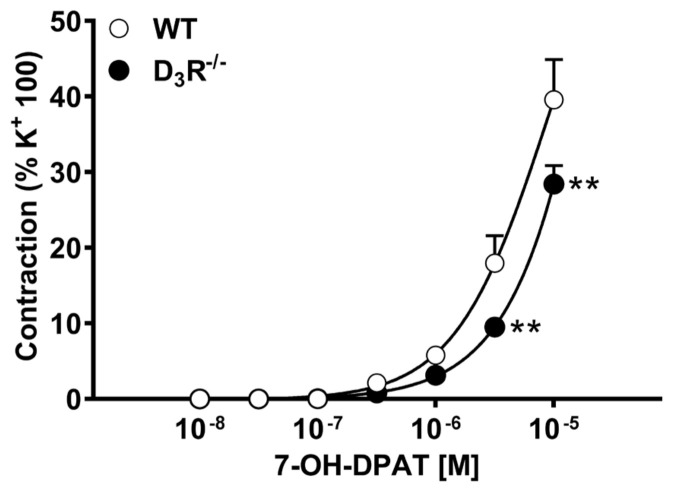
Vasoconstriction induced by 7-hydroxy-2-dipropylaminotetralin (7-OH-DPAT), in aortic segments isolated from wild-type (WT) and D3R^−^/^−^ mice. Each curve (with vertical bars representing standard errors) represents the average from 8–10 preparations. Two-way ANOVA (genotype: F_(1,294)_ = 13.4, *p* = 0.0003; agonist conc.: F_(6,294)_ = 108, *p* < 0.0001; genotype x agonist conc. F_(6,294)_ = 3.55, *p* = 0.0021) and Bonferroni’s test (** *p* < 0.01 vs. WT).

Preparations were subsequently exposed to cumulative concentrations of the D3R agonist 7-hydroxy-2-dipropylaminotetralin (7-OH-DPAT). As shown in Figure 2, 7-OH-DPAT induced a concentration-dependent contractile response in aorta from both WT and D3R^−^/^−^; this contraction was, however, weaker than that induced by PE, barely reaching 40–50% of KCl-induced contraction. The maximum effect was clearly not yet reached with 10 μM 7-OH-DPAT; however, we did not test higher concentrations because, based on the nanomolar affinity of 7-OH-DPAT for dopamine receptors reported in literature [37], the specificity of the effect at such high concentrations would had been quite questionable. The contractile responses induced by 7-OH-DPAT in preparations from D3R^−^/^−^ were smaller than in preparations from WT. This difference was statistically significant at 3 and 10 μM (*p* < 0.01).

**Figure 3:**
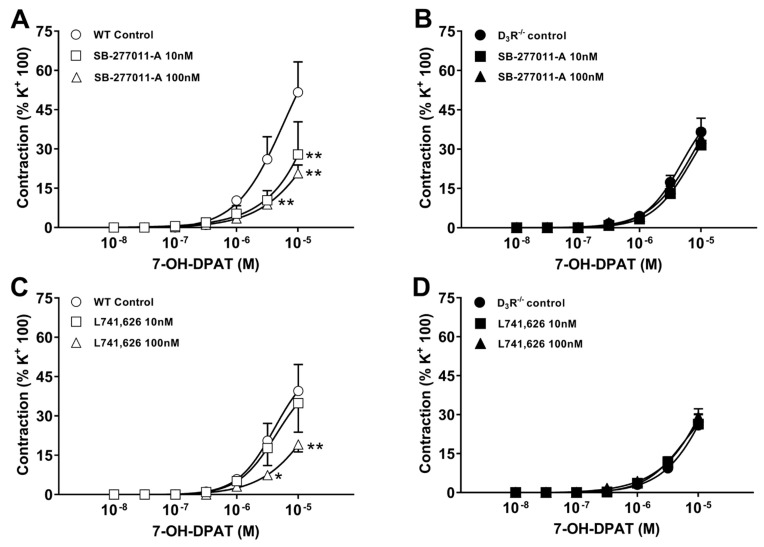
Effect of dopamine receptor antagonists on 7-hydroxy-2-dipropylaminotetralin (7-OH-DPAT)-induced vasoconstriction, in aortic segments isolated from wild-type (WT, **A**,**C**) and D3R^−^/^−^ mice (**B**,**D**). (**A**,**B**) Effect of the D3R antagonist SB-277011-A; (**C**,**D**) effect of the D2R antagonist L741,626. Each curve (with vertical bars representing standard errors) represents the average from 8–10 preparations. Two-way ANOVA, Panel A (antagonist conc.: F_(2,84)_ = 7.23, *p* = 0.0013; agonist conc.: F_(6,84)_ = 26.41, *p* < 0.0001; antagonist conc. x agonist conc. F_(12,84)_ = 2.50, *p* = 0.0076); Panel C (antagonist conc.: F_(2,105)_ = 3.53, *p* = 0.033; agonist conc.: F_(6,105)_ = 26.80, *p* < 0.0001); Panel A (antagonist conc.: F_(2,84)_ = 7.23, *p* = 0.0013; agonist conc.: F_(6,84)_ = 26.41, *p* < 0.0001; antagonist conc. × agonist conc. F_(12,105)_ = 1.16, *p* = 0.3163) and Bonferroni’s test (* *p* < 0.05, ** *p* < 0.01 vs. control).

In order to further analyze the D2R-like receptor subtype involved in the contractile response induced by 7-OH-DPAT, the aortic segments were pre-incubated with D3R and D2R receptors antagonists. In aortic segments from WT, control mice, incubation with either the selective dopamine D3R receptor antagonist SB-277011-A (10, 100 nM), or with the preferential dopamine D2R receptor antagonist L741,626 (10, 100 nM) reduced the contractile responses to 7-OH-DPAT (Figure 3A,C); SB-277011-A was significantly more potent than L741,626, attaining already a maximum inhibition at 10 nM. In contrast, in preparations from D3R^−^/^−^, neither SB-277011-A nor L741,626 significantly changed the contractile response to 7-OH-DPAT (Figure 3B,D). This set of experiments indicated that the contractile response to 7-OH-DPAT sensitive to the antagonism by SB-277011-A or L741,626 was attributable to a stimulation of D3R, because it was absent in the absence of D3R.

### 3.3. Epression of DR mRNA in Aortic Segments in Basal Condition and Following Knockdown

**Figure 4:**
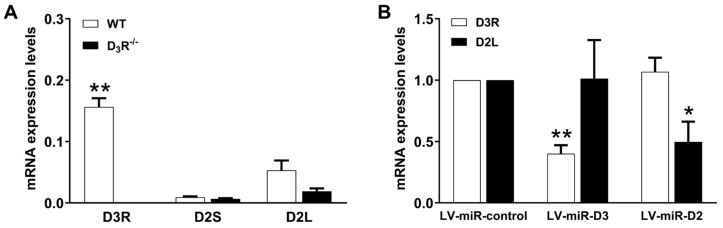
(**A**) Levels of mRNA encoding D3R and D2R (short- and long-splice variants, D2S, D2L) in aortic segments from wild-type (WT) and D3R^−^/^−^ mice. Total RNA was reverse transcribed; the corresponding cDNA was quantitated by real-time PCR. Levels of mRNA are normalized against the housekeeping gene GAPDH. Each column (with vertical bars representing standard errors) represents the average from 7–15 different RNA samples. Two-way ANOVA, (genotype: F_(1,59)_ = 75.2, *p* < 0.0001; mRNA expr.: F_(2,59)_ = 37.29, *p* < 0.0001; genotype × mRNA expr. F_(2,59)_ = 46.88, *p* = 0.0001) and Bonferroni’s test (** *p* < 0.01 vs. D2S and D2L). (**B**) Levels of mRNA encoding D3R and D2R in aortic segments from wild-type treated with lentiviral vectors, delivering synthetic miRNA (LV-miR-control, scrambled sequence; LV-miR-D3, targeting D3R; LV-miR-D2, targeting D2R). Total RNA was reverse transcribed; the corresponding cDNA was quantitated by real-time PCR. Levels of mRNA are normalized against the housekeeping gene GAPDH and the mRNA receptor expression in LV-miR-control. Each column (with vertical bars representing standard errors) represents the average from 5–9 different RNA samples. Two-way ANOVA, (LV-miR: F_(2,30)_ = 2.21 *p* = 0.12; mRNA expr.: F_(1,30)_ = 0.01, *p* = 0.90; LV-miR × mRNA expr. F_(2,30)_ = 9.39, *p* = 0.0007) and Bonferroni’s test (* *p* < 0.05, ** *p* < 0.01 vs. LV-miR-control).

To evaluate the expression levels of D3R and D2R receptors in aortic segments, we measured the mRNA levels of two different splicing variants of D2R dopamine receptors (long and short; D2L and D2S) and of D3R by RT-PCR. As shown in Figure 4A, the expression of D3R mRNA was significantly higher than either D2R variant. Moreover, no differences were observed in the mRNA levels of D2R between control and D3R^−^/^−^, confirming that the effect of 7-OH-DPAT on contractile response was due to the presence of D3R and not to D2R. Finally, as expected, D3R mRNA was not detectable in D3R^−^/^−^.

Aortic segments were exposed, in vitro, to lentiviruses carrying synthetic miRNA specific for D2R and D3R, to assess their contribution to contraction following their knockdown (see below). The expression level of DR mRNA was determined by RT-PCR, following lentivirus-miRNA-induced knockdown, and confirmed a specific knockdown, below 50% of basal levels, as shown in Figure 4B.

### 3.4. Effect of DR Knockdown on Vasomotor Responses to DR Agonist

**Figure 5:**
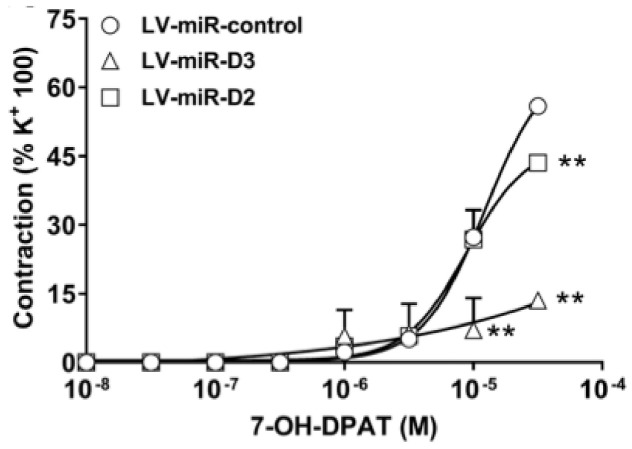
Vasoconstriction induced by 7-hydroxy-2-dipropylaminotetralin (7-OH-DPAT), in aortic segments from wild-type treated with lentiviral vectors delivering synthetic miRNA (LV-miR-control, scrambled sequence; LV-miR-D3, targeting D3R; LV-miR-D2, targeting D2R). Each curve (with vertical bars representing standard errors) represents the average from 6 preparations. Two-way ANOVA, (LV-miR: F_(2,56)_ = 12.91 *p* < 0.0001; agonist conc.: F_(7,56)_ = 75.17, *p* < 0.0001; LV-miR × agonist conc. F_(14,56)_ = 7.90, *p* < 0.0001) and Bonferroni’s test (** *p* < 0.01 vs. LV-miR-control).

The contribution of specific DR receptor subtypes to 7-OH-DPAT-induced vasoconstriction was further investigated in aortic segments where DR were knocked down by lentiviral delivery of synthetic miRNA. As shown in Figure 5, in preparations receiving miRNA directed to D2R (LV-miR-D2), 7-OH-DPAT induced contractions similar to those induced in preparations incubated with the scrambled control, except at the highest concentration tested (30 μM), where there was a mild but statistically significant reduction. In contrast, in preparations receiving miRNA directed to D3R, 7-OH-DPAT induced only weak contractions, barely attaining 15% of the KCl 100-induced ones and largely below both those in control and in LV-miR-D2 group.

## 4. Discussion

Despite the large amount of data available on DR in CNS, both in terms of expression and function in the context of DA transmission, less is known on DR in periphery [38]. In arteries, DA may induce vasoconstriction or, in certain districts, vasodilatation, which seems to be endothelium-dependent [38]. Taking advantage of the experience and tools available in our lab to study D3R, particularly the D3R null mouse colony (D3R^−^/^−^), we tested the hypothesis that D3R subtype may induce vasomotor effects in isolated mouse aorta. We first analyzed the vasomotor responses unrelated to DA, to characterize the functional baseline of our model. The vasoconstriction induced by KCl and PE as well as the vasodilatation to ACh were not different in WT and D3R^−^/^−^, indicating that our system was suitable for assessing vasomotor effects specifically related to D3R. We then tested the vasomotor effect of the selective D3R agonist 7-OH-DPAT [37], in WT and D3R^−^/^−^. This compound evoked concentration-dependent contractile responses not only in preparations from WT, but also in preparations from D3R^−^/^−^, albeit significantly weaker. This observation indicated that the contractile effect of 7-OH-DPAT was attributable in part to D3R stimulation, in part to other, undefined, receptors. Generally speaking, agonists show less selectivity than antagonists. In particular, molecular modeling as well as radioligand binding studies report 7-OH-DPAT as having a 20-100-fold selectivity for D3R over D2R [37]; thus, conceivably, at high concentrations, 7-OH-DPAT would bind other receptors able to induce contractile responses in vascular smooth muscle cells, possibly including adrenergic and 5-HT receptors. Worthy of mention, 5-HT receptors are known to share the highest number of multitarget ligands with other receptor subtypes, including about two thousand ligands with DR [39]. Table 1 reports the affinity for D2R, D3R, and 5-HT receptors of the ligands used in the present study.

We then further analyzed the DRs involved in 7-OH-DPAT-induced contraction by testing two antagonists, L741,626 and SB-277011-A, respectively, selective for D2R (about 16-fold, [37,44,45,46]) and D3R (about 100-fold, [47]). When tested in preparations from WT, both antagonists were able to significantly reduce the contractile response to 7-OH-DPAT, SB-277011-A resulting as more potent than L741,626; however, neither L741,626 nor SB-277011-A significantly affected the 7-OH-DPAT-induced contractions in preparations from D3R^−^/^−^. This latter observation further supports the view that part of contractile response induced by 7-OH-DPAT in mouse aorta is due to D3R stimulation, while another part, persistent in the presence of the D3R antagonist and in mice not expressing functional D3R, is unrelated to D3R. Notice that L741,626, considered a D2R antagonist, was also able to inhibit 7-OH-DPAT-induced contraction, but in WT only; this suggests that the inhibition by L741,626 occurred as a result of D3R antagonism, which is not surprising, based on the reported affinity of L741,626 for D2R and D3R.

We then tried to estimate the abundance of DR subtypes in mouse aorta. However, because the available antibodies do not clearly discriminate between D2R and D3R, which also have close molecular size, making it difficult to resolve them in SDS-PAGE, we used RT-qPCR to estimate DR mRNA abundance. D3R appeared about 5-fold more abundant than D2R in samples from WT mRNA and, as expected, was undetectable in aorta from D3R^−^/^−^. Furthermore, no differences were observed in D2R mRNA expression between WT and D3R^−^/^−^, ruling out potential compensatory D2R expression associated with D3R genetic deletion. Following transduction with lentivirus (72-h incubation), delivering synthetic microRNAs to specifically inactivate D2R (LV-miR-D2) or D3R (LV-miR-D3), the contractile response to 7-OH-DPAT was unaffected by LV-miR-D2, while it was significantly reduced by LV-miR-D3. These data, showing that variation in D3R mRNA expression is linked to the functional response induced by 7-OH-DPAT, are entirely consistent with data comparing functional responses in aorta from WT and D3R^−^/^−^. Again, the residual vasoconstriction elicited by high-concentration D3R agonist in D3R^−^/^−^ and/or in the presence of D3R antagonist is likely to be unrelated to DRs. We did not try to analyze the DR antagonism according to Schild [48], because a substantial part of the vasoconstriction elicited by our agonist, 7-OH-DPAT, appeared to be mediated through multiple receptors, including non-DR, being resistant to D2R antagonist, to D3R antagonist and/or to D3R genetic deletion. This part of the response to 7-OH-DPAT, which, per se, was not so robust (about 50% of KCl 100 mM-induced contraction), made impossible an accurate Schild’s analysis. Indeed, in our system, DR-independent effects of 7-OH-DPAT might occur already at concentrations beyond 1 μM, which was the threshold of 7-OH-DPAT contractile effect.

DRs have been identified so far in several arteries, such as aorta, renal, coronary, pulmonary, mesenteric, and cerebral arteries of different species, including humans [24]. Furthermore, they have been implicated in the patho-physiology of diseases, such as hypertension, atherosclerosis, diabetes, and obesity, as well as in the cardiovascular and metabolic side effects associated with the use of antipsychotics [49]. Some reports suggest that D2R-like receptors improves the metabolic profile and decreases the systolic blood pressure [50,51,52], proposing the D2R-like receptors as a potential therapeutic target in diabetes and hypertension. In contrast, a decrease in D1R, D2R, and D5R expression in mesenteric arteries has been related to increase in blood pressure [53,54].

Identification of DR in vessels was initially carried out through functional and/or radioligand binding studies, using agonists and antagonists not specific and selective enough to provide univocal and unambiguous conclusions. More recently, DR expression in arteries has been assessed at the mRNA and/or at the protein level. While mRNA expression does not directly correspond to the receptor protein in the plasma membrane, at least it provides an estimate of the relative abundance of different subtypes, with the advantage of absolute specificity, not afforded by pharmacological studies. In contrast, antibodies do not resolve the different DR proteins on the cell surface, nor do they in immunoblots, because the molecular size of DR subtypes is relatively close [2]. Data on DR mRNA and/or protein expression in vessels generally indicate D1R-like receptors expressed in endothelium and D2R-like receptors expressed in prejunctional sympathetic nerve endings, while less is known on vascular smooth muscle cells, where presumably DRs do not reach an expression level comparable to that of endothelium and/or of the surrounding sympathetic nerve endings [38]. At variance with these earlier studies, here we provided three different lines of data, from pharmacology, gene deletion, mRNA expression and knockdown, consistent and converging to D3R as mediating vasoconstriction in isolated mouse aorta. Interestingly, since DA has been detected in endothelium from some district [26], we speculate that endothelium may serve, in vivo, as a DA source to exert autocrine (on endothelium itself) and/or paracrine (stimulation of DRs on vascular smooth muscle cells) effects. Stimulation of D1R in endothelium is thought to induce nitric oxide release and vasodilatation [26,38]. In the present study we did not analyze D1R-mediated effects, because they are reported in literature and clearly converge to vasodilatation. Worthy of note, DA-induced vasodilatation is considered as mostly mediated by D1R expressed in smooth muscle cells. In fact, in renal arteries, D1R located on smooth muscle cells mediates the vasodilatation induced by fenoldopam, a D1R agonist. In particular, D1R has been detected at the protein level (immunoblot) in renal arteries [30,55], where it induces vasodilatation through the Gs/cyclic AMP/Protein kinase A-signaling pathway [31,32,55].

Psychiatric patients seem to be at increased risk for cardiovascular events, such as 2- to 5-fold greater risk of coronary heart disease and a 2- to 3-fold greater risk of cardiac mortality [56,57,58]. A more recent study [59] suggests that antipsychotic use may be associated with acute myocardial infarction, possibly related to D3R blockade. Our data provide evidence about DR subtypes involved in arterial constriction which do not corroborate this view. However, the in vivo situation following chronic treatment is obviously much more complex, and has to take into account a number of relevant effects on other districts (for example, endocrine pancreas, insulin release and insulin resistance, and effects on heart electrophysiology, including effects on QT interval), which exert a relevant impact on cardiovascular patho-physiology [38].

In this context, understanding the direct effect of DR stimulation in critical arterial districts, such as the cerebral and the coronary ones, may not only help in improving the safety of chronic drug treatments acting on DRs, but also define novel pharmacological targets to treat ischemic conditions.

## 5. Conclusions

In conclusion, our data indicate that, at least in mouse aorta, D3R stimulation induces vasoconstriction, while D2R stimulation does not exert significant vasomotor effects. These functional data are consistent with mRNA expression assessment and knockdown, showing higher D3R expression in WT aortic tissues and reduction of contractile responses following D3R knockdown. The residual vasoconstriction elicited by high concentration D3R agonist in D3R^−^/^−^ and/or in the presence of D3R antagonist is likely to be unrelated to DR stimulation. Additional studies are needed to determine whether or not D3R receptors contribute to vascular tone in other vascular beds/species. Furthermore, the endogenous agonist responsible for the stimulation of these D3R remains elusive, given the negligible amount of DA available in periphery, mostly spilling from sympathetic nerve endings and/or adrenal medulla, though some may also come from endothelium. These data may be useful to understand the safety of drug treatments acting on DRs and/or to find novel drug targets for cardiovascular diseases.

## Figures and Tables

**Table 1:** Reported affinity (Ki, nM) of 7-OH-DPAT, SB-277011-A, and L741,626 for D2R, D3R, and 5-HT receptors; in square brackets the reference.

	D2R				D3R				5-HT_1A_	5-HT_1D_	5-HT_2A_	5-HT_2B_
**7-OH-DPAT**	2.6–165 [40]	142 [41]	60 [42]	103 [43]	0.4–1.2 [40]	2.90 [41]	1.6 [42]	2.1 [43]	72.7 [41]			
**L741,626**	11.2 [44]	2.4 [45]	4.0–6.3 [46]		163 [44]	100 [45]	63 [46]				316 [46]	631 [46]
**SB-277011-A**	1047 [47]				11.0 [47]					1621 [47]		1288 [47]
